# Direct measurement of free glucocorticoids in small volumes of mouse and rat serum using ultrafiltration and liquid chromatography-tandem mass spectrometry

**DOI:** 10.1371/journal.pone.0341089

**Published:** 2026-01-22

**Authors:** Anna Mazurenko, Melody Salehzadeh, Kiran K. Soma

**Affiliations:** 1 Department of Psychology, The University of British Columbia, Vancouver, British Columbia, Canada; 2 Djavad Mowafaghian Centre for Brain Health, The University of British Columbia, Vancouver, British Columbia, Canada; 3 Department of Zoology, The University of British Columbia, Vancouver, British Columbia, Canada; 4 Graduate Program in Neuroscience, The University of British Columbia, Vancouver, British Columbia, Canada; Tshwane University of Technology, SOUTH AFRICA

## Abstract

Glucocorticoids are critical steroid hormones secreted from the adrenal glands. In mice and rats, over 90% of circulating corticosterone is bound to proteins such as corticosteroid-binding globulin and albumin, and the rest is unbound (free). Only free glucocorticoids can enter cells and bind receptors, so it is crucial to measure free glucocorticoids. Some studies have estimated free glucocorticoid levels, but such estimates might be inaccurate as they do not take temperature and protein binding by competing steroids into account. Far fewer studies have directly measured free glucocorticoid levels in serum, and current methods to do so are time-consuming and require sample volumes (200 + µl) that are difficult to obtain from mice. Here, we developed a method to directly measure free glucocorticoids in a small volume of rodent serum using ultrafiltration and liquid chromatography-tandem mass spectrometry. We validated this method to measure free 11-deoxycorticosterone (corticosterone precursor), corticosterone, and 11-dehydrocorticosterone (corticosterone metabolite) in as little as 30 µl of mouse and rat serum. Ultrafiltration produces results that are qualitatively similar to those from equilibrium dialysis, an established method that requires a much larger sample volume (200 + µl). We then applied our novel method to examine the effects of lipopolysaccharide (LPS), an immune stressor that is known to increase total corticosterone levels, free corticosterone levels, and percent free corticosterone. We administered saline vehicle or LPS to adult male and female mice, collected blood 4 hr later, and measured total and free glucocorticoid levels in serum from individual mice. As expected, LPS increased total and free corticosterone levels and percent free corticosterone. This simple, robust, and rapid method allows direct measurement of free corticosterone and other steroids in 30 µl of rodent serum.

## Introduction

Glucocorticoids (GCs) are steroid hormones that are regulated by the hypothalamic-pituitary-adrenal (HPA) axis and act on nearly all tissues. GCs have important roles in metabolism [1], immunity [2] and brain function [3]. Dysregulated GC levels can lead to serious conditions, such as Cushing’s and Addison’s diseases [4], cardiovascular disease [4], and psychiatric illnesses [5]. Exogenous GCs are commonly used for treating metabolic and inflammatory conditions [6,7].

Corticosterone is the main adrenal GC in many rodents, such as mice and rats, and binds with high affinity to corticosteroid-binding globulin (CBG). CBG is produced in the liver and released into blood [8,9]. In rodent serum, 80–90% of corticosterone is bound to CBG, 5–10% of corticosterone is bound to other proteins (e.g., albumin), and the rest (less than 10%) is unbound (free), based on equilibrium dialysis and other methods [10–13]. While CBG-bound corticosterone is inactive, free corticosterone can enter cells and bind to glucocorticoid and mineralocorticoid receptors [14,15]. Following a stressor, the adrenal glands secrete corticosterone, and total (bound + free) corticosterone levels rise. Circulating CBG levels appear to increase or decrease after a stressor, depending on stressor severity and duration [16–18], which allows fine-tuning of free corticosterone levels. Dysregulation of CBG is associated with hypotension, fatigue, and immune deficits [19–21].

In rodents, CBG can also bind 11-deoxycorticosterone (DOC; a corticosterone precursor), progesterone (a DOC precursor), and 11-dehydrocorticosterone (DHC; a corticosterone metabolite), albeit with lower affinity than for corticosterone. These other steroids, when not bound to CBG, can enter tissues and be locally converted to corticosterone within some tissues [22]. Moreover, free progesterone can bind to progesterone receptors. DOC, progesterone, and DHC can bind to CBG and compete with corticosterone (e.g., during pregnancy, when progesterone levels are high) [22–28]. Thus, free DOC, progesterone, and DHC levels can affect local corticosterone levels via local production, and these steroids compete with corticosterone for binding to CBG.

Total and free GC levels in serum increase in response to immune stressors [29]. A common immune stressor is administration of lipopolysaccharide (LPS), an endotoxin on gram-negative bacteria (e.g., *E. coli*), which increases total corticosterone levels [30–33] but decreases CBG levels [18,34,35] and increases cleavage of CBG [36] in rodents. Overall, LPS treatment is known to increase free corticosterone levels and the percent free corticosterone [18,34,37].

Few studies have directly measured free corticosterone levels, and even fewer have directly measured free progesterone, DOC, or DHC levels in rodent serum [23,38,39]. Most rodent studies only measure total corticosterone levels in serum. Some studies have *estimated* free corticosterone levels using a model that includes total corticosterone level, CBG capacity, and CBG affinity for corticosterone (typically measured at a non-physiological temperature, such as 4°C) [14,40,41]. The estimation of free corticosterone levels using such models is potentially inaccurate because: 1) CBG affinity for corticosterone is sensitive to temperature [42,43], 2) other proteins, such as albumin, bind corticosterone with high capacity and low affinity [16,44–46], and 3) other steroids, such as progesterone, compete with corticosterone for binding to CBG [47]. It is possible that free GC levels are not often directly measured because existing methods are complex, time consuming, and require large volumes of serum. For example, equilibrium dialysis can be used to directly measure free GC levels, but it requires ~24 hr and 200 + μl of serum [23,48,49], which is challenging to collect from mice and young rats and often necessitates pooling samples among individuals.

This study describes a simple, robust, and rapid method to directly measure free corticosterone, DOC, and DHC levels in a small amount (30 µl) of mouse and rat serum. We used ultrafiltration, followed by an accurate, precise, specific, and sensitive liquid chromatography-tandem mass spectrometry (LC-MS/MS) assay to measure multiple steroids in ultrafiltrates. Ultrafiltration separates large molecules from small molecules, such as CBG-bound corticosterone (50–60 kDa [50]) from free corticosterone (346 Da). We validated the use of ultrafiltration with as little as 30 µl of mouse serum (Study 1), compared ultrafiltration with a well-established method (equilibrium dialysis) using pooled serum from vehicle (VEH)- and LPS-treated mice (Study 2), and compared ultrafiltration with equilibrium dialysis using pooled rat serum (Study 3). Finally, we applied the method to quantify free steroid levels in serum from individual vehicle (VEH)- and LPS-treated mice, as a proof of principle and to demonstrate our method’s performance using samples that reflect biological variation (Study 4).

## Materials and methods

### General protocols

The protocol described in this peer-reviewed article is published on protocols.io, dx.doi.org/10.17504/protocols.io.5qpvodkddg4o/v1 and is included for printing as Supporting Information File 1 with this article.

#### Sample collection.

Mice and rats were rapidly and deeply anesthetized using 5% isoflurane in oxygen (2 L/min) and euthanized by rapid decapitation. Trunk blood was collected within 3 min of cage disturbance (to limit the effect of stress on steroid levels [51]) in 1.5 ml polypropylene microcentrifuge tubes (VWR, Edmonton, AB), and blood samples were kept on wet ice for < 3 hr. Blood samples were centrifuged at 5,000 g for 10 min at 4°C, and serum was transferred to 1.5 ml polypropylene microcentrifuge tubes and stored at –70°C. Tissues (brain, lymphoid organs) were collected from animals for a separate, independent study, and thus, animals were euthanized at the end of experiment before reaching humane endpoint.

The procedures complied with the Canadian Council on Animal Care, and protocols received approval from The University of British Columbia Animal Care Committee (Protocols A23-0023 and A22-0191). All researchers were trained and received required approval for conducting the procedures by UBC Animal Care Services.

### Ultrafiltration

**Fig 1:**
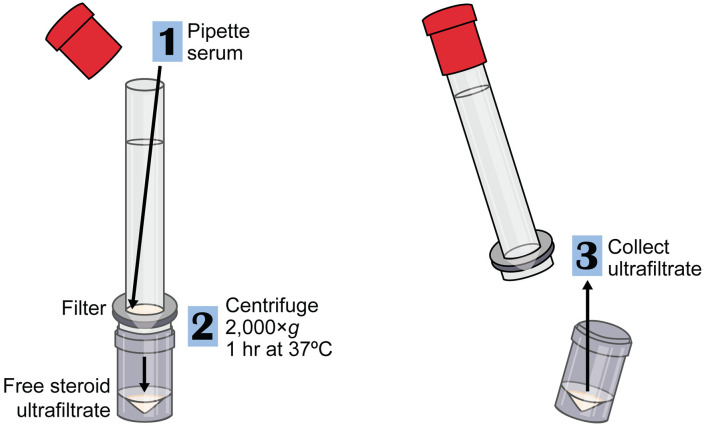
Ultrafiltration protocol. Steps for ultrafiltration with Centrifree Ultrafiltration Device with Ultracel PL membrane to isolate free steroids from serum.

We used the Centrifree Ultrafiltration Device with Ultracel PL membrane with a 30 kDa molecular weight cut-off (Catalog #4104, Millipore Sigma-Aldrich, Oakville, Ontario, Canada) (Fig 1). First, we thawed serum samples gently on wet ice, and then warmed them to 37°C in a water bath for 10 min. Then we added serum (30–150 μl) at a 45° angle to ultrafiltration devices and replaced the caps on the devices. We centrifuged the ultrafiltration devices in a pre-warmed centrifuge (Avanti J-15R, Beckman Coulter) at 2,000 g at 37°C for 1 hr. We transferred the ultrafiltrate into 1.5 ml polypropylene microcentrifuge tubes (VWR, Edmonton, AB) and stored ultrafiltrate samples at –70°C.

### Steroid extraction

We thawed samples on wet ice, transferred the desired volume into 2 ml polypropylene screw cap tubes with 5 ceramic zirconium oxide beads, and added 50 µl of deuterated internal standards (progesterone-d9, corticosterone-d8, C/D/N Isotopes Inc., Pointe-Claire, Canada) in 50:50 High-Performance Liquid Chromatography (HPLC)-grade methanol:MilliQ water to track steroid recovery. Progesterone-d9 was used to track recovery for progesterone, and corticosterone-d8 to track recovery for DOC, corticosterone, and DHC. We precipitated proteins and extracted steroids by adding 1 ml HPLC-grade ethyl acetate (Thermo Fisher Scientific, Burnaby, BC) and homogenizing samples with a bead mill homogenizer at 4 m/s for 30 s. We centrifuged samples at 16,100 g for 5 min and transferred 1 ml of the supernatant to a pre-cleaned borosilicate glass culture tube. We added 500 µl MilliQ water to each tube, vortexed, and centrifuged at 3,200 g for 2 min. We transferred the ethyl acetate to a new culture tube and discarded the MilliQ water (containing trace amounts of glycerine from the ultrafiltration device). We fully dried samples in a water bath at 60°C under a gentle stream of nitrogen gas for 5–10 min. We resuspended dried residues in 200 µl of 25% HPLC-grade methanol:MilliQ water, vortexed, and centrifuged at 3,200 g for 1 min. We transferred the supernatant to a 0.6 ml polypropylene microcentrifuge tube (VWR, Edmonton, AB), centrifuged at 16,100 g for 2 min, transferred 150 μl of the supernatant to a glass LC vial insert, and stored samples at −20°C.

### Steroid analysis by LC-MS/MS

Progesterone, DOC, corticosterone, and DHC levels were quantified by LC-MS/MS as previously described [52]. Samples were loaded into an autosampler at 15°C. Then 35 μl was injected into a Nexera X2 UHPLC system (Shimadzu Corp., Japan), passed through an in-line filter, a SecurityGuard^™^ ULTRA C18 UHPLC guard column (2.1 mm) (Phenomenex), and a Kinetex^®^ Core-shell C18 column (2.1 x 50 mm; 2.6 μm; at 40 °C) (Phenomenex) with a gradient binary mobile system using 0.1 mM ammonium fluoride in MilliQ water (mobile phase A) and HPLC-grade methanol (mobile phase B). The flow rate was 0.4 ml/min. Mobile phase B was at 10% for 0.5 min during loading, and then the gradient profile began at 42% mobile phase B for 3.5 min before being increased to 60% mobile phase B until 9.4 min. The gradient was 60%−70% mobile phase B from 9.4 to 9.5 min, then increased to 98% mobile phase B until 11.9 min, and then a column wash at 98% mobile phase B until 13.4 min. The mobile phase B was returned to starting conditions for 1 min. Total run time was 14.9 min. Before and after each injection, the injection needle was rinsed with HPLC-grade methanol.

Steroid quantification was performed on Sciex 6500 Qtrap triple quadrupole tandem mass spectrometer (Sciex LLC, MA). Steroids were quantified with scheduled multiple reaction monitoring (sMRM) with two mass transitions per analyte and one mass transition per internal standard. Positive electrospray ionization was used for all steroids. Samples were processed alongside calibration curves, blanks (with internal standards), double blanks (without internal standards), and quality controls (2 and 200 pg of steroids). The calibration curve range was 0.4–1000 pg (Study 1), 0.4–2000 pg (Study 2), 0.2–4000 pg (Study 3), and 0.2–2000 pg (Study 4), made in 50:50 HPLC-grade methanol:MilliQ water. Quality controls were measured in triplicate in each assay.

### Study 1 – determining minimum volume of mouse serum required

We assessed free steroid levels in serum volumes from 30 to 150 µl, following ultrafiltration. We tested 5 volumes (30, 60, 90, 120, 150 µl) using a pool of mouse serum.

#### Animals.

Subjects were adult (post-natal day (PND) 65+) male and female C57BL/6J mice. Mice were housed at the Centre for Disease Modeling at The University of British Columbia under 20–22°C, 40–70% relative humidity, and a 14:10 light:dark cycle (lights on 0600–2000 hr). Mice were group-housed (2–5 same-sex mice/cage) in ventilated Ehret polysulfone Type IIL cages with a CO_2_ membrane, beta-chip bedding, and free access to water (purified by reverse osmosis and sterilized by chlorination) and food (Harland Teklad Global diet 2918). For enrichment, mice were provided with a red translucent hut and crinkle paper. Health states of mice were monitored daily by animal technicians.

### Ultrafiltration

Serum samples were thawed on wet ice, and then a pool of mouse serum (2.5 ml, from n = 8 animals, males and females) was created. The pool of serum was divided into 5–6 replicates of 5 volumes of serum: 30, 60, 90, 120, and 150 μl. Each serum sample was added to a separate ultrafiltration device. The ultrafiltration protocol was followed as described above. Ultrafiltrates were stored at –70°C until steroid extraction.

### Study 2 – comparing ultrafiltration and equilibrium dialysis using mouse serum

We compared ultrafiltration and equilibrium dialysis techniques to measure free steroids in pooled mouse serum. Equilibrium dialysis is a well-known method for measuring free steroids [23,48,49], but it requires 6–7 × more serum than ultrafiltration. We measured free steroids in two pools of serum (from VEH- or LPS-treated mice) by using ultrafiltration with 30 µl aliquots of serum and equilibrium dialysis with 200 µl aliquots of serum.

#### Animals.

Subjects were C57BL/6J mice, housed as described in Study 1. Adult mice (~PND90) were injected (i.p.) with either sterile nonpyrogenic 0.9% saline VEH or LPS (400 μg/kg bw). Both solutions were warmed on a heating pad before injection, and all injections were administered between 0900–1100 hr. Mice (2–3 littermates per cage) were injected concurrently using 27G needles and then returned to their cage and left undisturbed until euthanasia 4 hr later. Subjects were randomly assigned to treatment and balanced for sex. Experimenters were not blinded to subject treatment during injections. Animals were monitored closely for 15 min and at 1 hr post treatment. All animals survived treatment. Four hr after injection, when GC levels are increased by LPS [53], mouse serum was collected as described above.

### Ultrafiltration and equilibrium dialysis

Serum samples were thawed on wet ice (n = 8–10/treatment/sex). One serum pool was created from VEH-treated mice, and another serum pool was created from LPS-treated mice. Each serum pool was split into 30 μl aliquots for ultrafiltration (n = 6 replicates/treatment) and 200 µl aliquots for equilibrium dialysis (n = 5–6 replicates/treatment). In addition, 5 µl aliquots for total steroid measurement were made (n = 5 replicates/treatment) and stored at –70°C.

The ultrafiltration protocol was followed as described above, and ultrafiltrates were stored at –70°C. Equilibrium dialysis was conducted as described before [23], except 200 μl of serum and phosphate-buffered saline (PBS) were used (instead of 250 μl). For each sample, the cap was cut off a 1.5 ml polypropylene microcentrifuge tube, and the bottom conical half of the tube was cut off. Then 200 μl PBS was added to the cap, and then the cap was covered with a PBS-equilibrated 3.5K MWCO dialysis membrane (Catalog #88244, Thermo Fisher Scientific). The microcentrifuge tube was placed upside-down onto the covered lid to secure the membrane. Undiluted serum (200 μl) was carefully added on top of the membrane via the open end of the microcentrifuge tube. The open end of the microcentrifuge tube was sealed with parafilm, and the samples were incubated overnight (~24 hr) at 37°C, to allow enough time to reach equilibrium at physiological temperature. The parafilm was removed, and the serum above the dialysis membrane was collected carefully to avoid puncturing the membrane and contaminating the dialysate. The devices were then very carefully disassembled, and 120 μl of dialysate, which contains free steroids, was collected from the microcentrifuge tube cap while avoiding contamination with serum. All samples were stored at –70°C until steroid extraction.

### Study 3 – comparing ultrafiltration and equilibrium dialysis using rat serum

We compared ultrafiltration and equilibrium dialysis techniques to measure free steroids in pooled rat serum. We measured free steroids in aliquots of pooled rat serum by ultrafiltration (30 µl aliquots) and equilibrium dialysis (200 µl aliquots).

#### Animals.

Subjects were adult (~PND200) male and female Long-Evans rats housed at the Centre for Disease Modeling at The University of British Columbia under a 12:12 light:dark cycle (lights on 0800–2000 hr). Rats were group housed in open-top cages with beta-chip bedding. For enrichment, rats were provided with a red translucent hut and paper towel. Health states of rats were monitored daily by animal technicians. Serum was collected as described.

### Ultrafiltration and equilibrium dialysis

Serum samples (n = 2/sex) were thawed on wet ice and pooled. The serum pool was split into 30 μl aliquots for ultrafiltration (n = 6 replicates) and 200 µl aliquots for equilibrium dialysis (n = 6 replicates). Also, 5 µl aliquots for total steroid measurement were made (n = 5 replicates) and stored at –70°C. The ultrafiltration and equilibrium dialysis protocols were followed as described above, and ultrafiltrate and dialysate samples were stored at –70°C until steroid extraction.

### Study 4 – application of ultrafiltration to measure the effects of LPS in individual mice

We used ultrafiltration with serum from *individual* VEH- and LPS-treated male and female mice (n = 9–10/group) to assess free steroid levels after an acute immune stressor.

#### Animals.

Subjects were C57BL/6J mice, housed as described in Study 1. The adult mice (PND65+) (n = 9–10/treatment/sex) were injected (i.p.) with either sterile nonpyrogenic 0.9% saline VEH or LPS (50 μg/kg bw). Both solutions were warmed on a heating pad before injection, and all injections were administered between 0900–1100 hr. Mice (2–3 littermates per cage) were injected concurrently using 27G needles and then returned to their cage and left undisturbed until euthanasia. Subjects were randomly assigned to treatment and balanced for sex. Experimenters were not blinded to subject treatment during injections. Animals were monitored closely for 15 min and at 1 hr post treatment. Four hr after injection, mouse serum was collected as described above.

### Ultrafiltration

Serum samples were thawed on wet ice. For each sample, 30 μl of serum was used for ultrafiltration, and 2–5 µl was used for total steroid measurement. The ultrafiltration protocol was followed as described above. Ultrafiltrates and serum samples were stored at –70°C until steroid extraction.

### Data analysis

A value was considered below the lower limit of quantification (LLOQ) for steroid analysis if neither transition was present. Free progesterone was below the LLOQ in all ultrafiltrates. For DOC and DHC, values below the LLOQ were imputed when at least 20% of samples in a group were above the LLOQ, using quantile regression imputation of left-censored missing data [54–56].

Free steroid concentration in the ultrafiltrate was calculated by dividing the amount of steroid in the ultrafiltrate by the volume of serum used. Free steroid concentration in the dialysate was calculated by dividing the amount of steroid in the dialysate by the volume of dialysate used for steroid extraction.

In Study 1, the effects of serum volume were examined using one-way analysis of variance (ANOVA). When there was a significant main effect of serum volume, Dunnett’s multiple comparisons post hoc analyses were conducted to compare the 30, 60, 90, and 120 μl groups to the 150 μl group. In Studies 1, 2, and 3, the percent coefficient of variance (%CV) of each group was calculated, with <20% CV considered acceptable [57]. In Studies 2 and 3, inferential statistics were not performed.

In Study 4, the main effects of Treatment and Sex and the Treatment × Sex interaction were examined using two-way ANOVAs. When there was a significant Treatment × Sex interaction, post hoc analyses were conducted, comparing all groups to each other using Tukey’s multiple comparisons test. Two-tailed Student’s or Welch’s t-tests were used to examine the effects of LPS on the percent free DOC, corticosterone, and DHC. Correlations between total levels and free levels of DOC, corticosterone, and DHC were examined using Spearman’s rho.

All data were analyzed using GraphPad Prism 10.1.1. except Spearman rho correlations were analyzed using GraphPad Prism 10.6.1. Statistical significance was set at *p* ≤ 0.05. Results are displayed as mean ± standard error of the mean (SEM). Data were visually inspected for normality using quantile-quantile, residual, and homoscedasticity plots. Data were log-transformed prior to analysis when necessary to ensure homogeneity of variance. All graphs depict non-transformed data. Experimenters were blinded to group during data analyses.

## Results

### Study 1 – determining minimum volume of mouse serum required

Ultrafiltration devices yielded consistent (<20% CV) ultrafiltrate volumes for each volume of mouse serum tested (%CV: 11.7% for 30 μl, 4.8% for 60 μl, 6.0% for 90 μl, 3.4% for 120 μl, and 12.0% for 150 μl). Ultrafiltration also generally yielded precise free DOC, corticosterone, and DHC levels for each volume of mouse serum tested, although the %CV for DOC in the 90 μl group was slightly more than 20% (Table 1). Progesterone was below the LLOQ in all ultrafiltrates.

**Table 1:** Precision (%CV) of free steroid measurement by ultrafiltration of mouse serum.

Serum Volume (μl)	DOC	Corticosterone	DHC
30	12.4	6.4	17.2
60	5.7	2.9	8.4
90	22.0	14.8	13.7
120	13.1	8.1	8.9
150	14.6	8.0	12.8

Serum from adult male and female mice (n = 8 mice) was pooled and then split into technical replicates (n = 5–6/volume) for ultrafiltration. Percent coefficient of variation (%CV) is shown. Abbreviations: DOC, 11-deoxycorticosterone; DHC, 11-dehydrocorticosterone.

**Fig 2:**
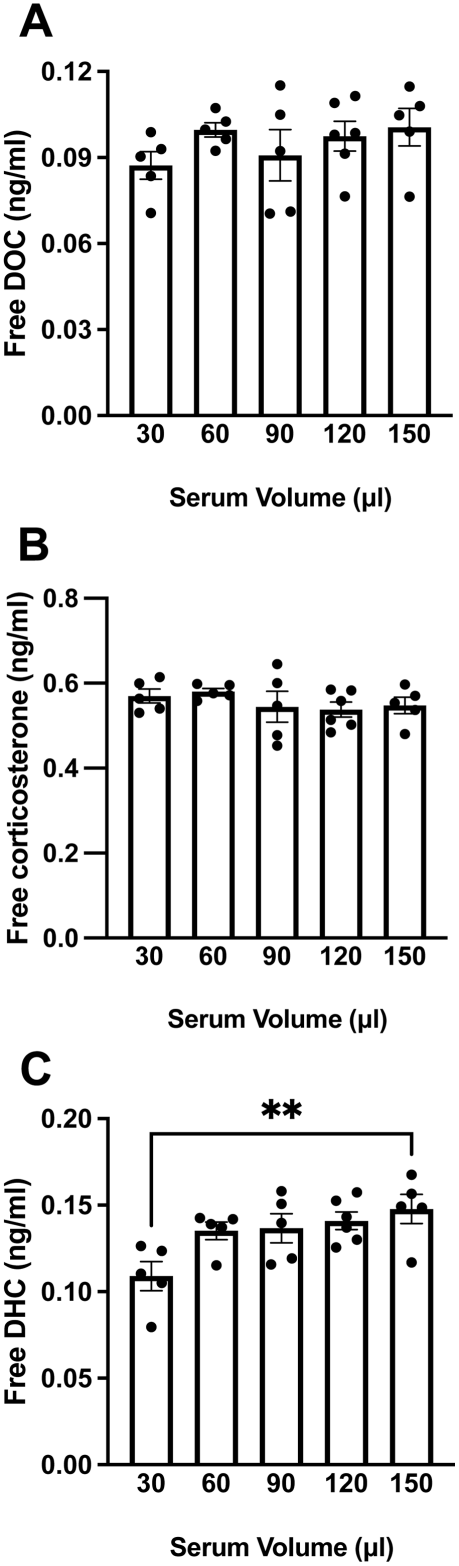
Free steroid measurement by ultrafiltration in a range of mouse serum volumes. (A) Free 11-deoxycorticosterone (DOC), (B) free corticosterone, and (C) free 11-dehydrocorticosterone (DHC) levels (ng/ml) in ultrafiltrate. Mouse serum was pooled (n = 8 mice) and then split into technical replicates (n = 5–6/volume) for ultrafiltration. Data are shown as mean ± SEM. Data were analyzed using one-way ANOVAs, and in case of a significant effect of volume, post hoc analyses were conducted using Dunnett’s multiple comparisons tests. **p ≤ 0.01.

Overall, free steroid levels were not significantly different across the volumes of mouse serum tested. There were no significant differences for free DOC (F(4,21)=0.93, p = 0.46; Fig 2A) and free corticosterone (F(4,21)=0.72, p = 0.59; Fig 2B). There was a significant main effect of serum volume for free DHC (F(4,21)=4.18, p = 0.01; Fig 2C), with lower free DHC in the 30 μl group than the 150 μl group (p = 0.004). Overall, free corticosterone level was higher than free DOC and DHC levels. In summary, 30 μl of mouse serum was acceptable for ultrafiltration.

### Study 2 – comparing ultrafiltration and equilibrium dialysis using mouse serum

With 30 µl mouse serum, ultrafiltration devices again yielded consistent ultrafiltrate volumes (8.7%CV). Both ultrafiltration and equilibrium dialysis generally had acceptable precision for free DOC, corticosterone, and DHC measurement in VEH and LPS mouse serum pools, although the %CV for DOC in the ultrafiltration VEH group was slightly more than 20% (Table 2).

**Table 2:** Precision (%CV) of free steroid measurement by ultrafiltration or equilibrium dialysis of mouse serum.

Group	DOC	Corticosterone	DHC
Ultrafiltration – VEH	21.1	13.7	10.8
Ultrafiltration – LPS	19.5	15.1	16.9
Dialysis – VEH	12.3	8.4	8.2
Dialysis – LPS	11.9	5.7	3.3

Mouse serum samples (n = 8–10 mice/treatment/sex) were collected 4 hr after an i.p. injection of saline vehicle (VEH) or 400 μg/kg bw lipopolysaccharide (LPS), combined into one VEH pool and one LPS pool, and then each pool was split into technical replicates (n = 5–6/treatment/method). Abbreviations: DOC, 11-deoxycorticosterone; DHC, 11-dehydrocorticosterone.

**Fig 3:**
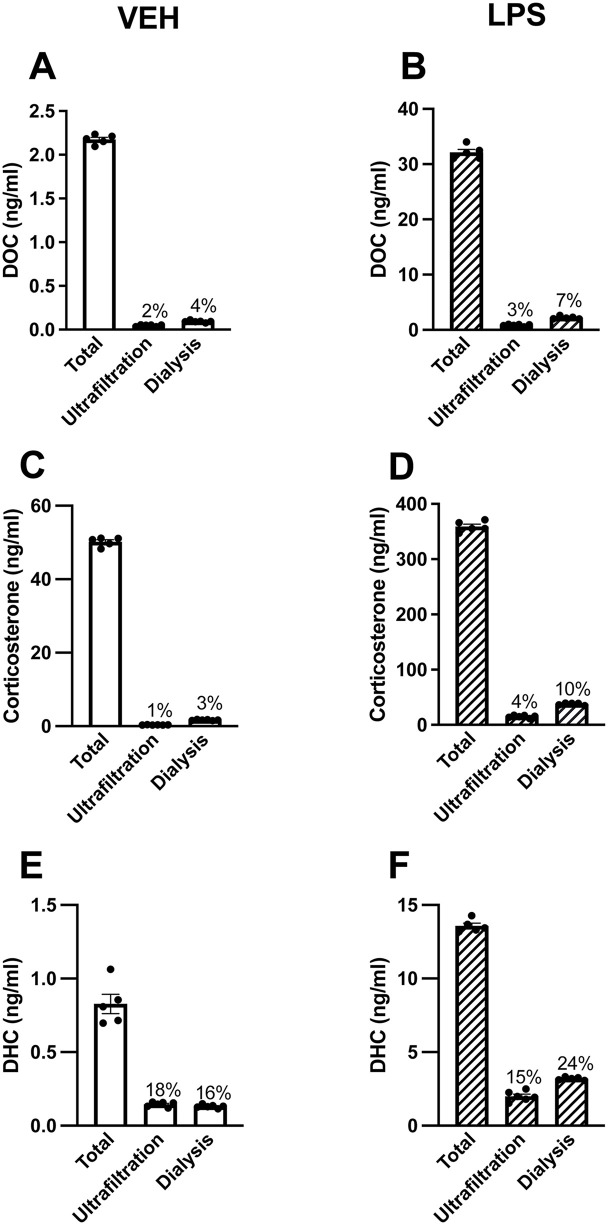
Free steroids measured via ultrafiltration and equilibrium dialysis in mice treated with vehicle or lipopolysaccharide. (A, B) 11-deoxycorticosterone (DOC), (C, D) corticosterone, and (E, F) 11-dehydrocorticosterone (DHC) total and free levels (ng/ml). Mouse serum samples (n = 8–10 mice/treatment/sex) were collected 4 hr after an i.p. injection of saline vehicle (VEH, open bars) or 400 μg/kg bw lipopolysaccharide (LPS, hatched bars). Samples were combined into one VEH pool and one LPS pool and then split into technical replicates (n = 5–6/treatment/method). Data are shown as mean ± SEM. The average percent free steroid is denoted above bars.

Free steroid levels (and the percent free steroid) in the mouse serum pools were generally similar with both methods, although slightly lower with ultrafiltration than equilibrium dialysis (Fig 3). Moreover, as expected, free GC levels were far lower than total GC levels with both methods. For example, in the VEH mouse serum pool, the percent free corticosterone was 1% using ultrafiltration and 3% using equilibrium dialysis (Fig 3C). In the LPS mouse serum pool, the percent free corticosterone was 4% using ultrafiltration and 10% using equilibrium dialysis (Fig 3D). With both methods, LPS administration increased the percent free corticosterone by approximately 3 × . Also, as expected, the percent free DHC was greater than the percent free DOC and corticosterone with both methods. Free progesterone levels were below the LLOQ in ultrafiltrates but not dialysates [58].

### Study 3 – comparing ultrafiltration and equilibrium dialysis using rat serum

With 30 µl rat serum, ultrafiltration devices again yielded consistent ultrafiltrate volumes (9.0%CV). Ultrafiltration and equilibrium dialysis both yielded precise free corticosterone and DHC levels in the rat serum pool (Table 3); DOC and progesterone were below the LLOQ in ultrafiltrates.

**Table 3:** Precision (%CV) of free steroid measurement by ultrafiltration or equilibrium dialysis of rat serum.

Method	Corticosterone	DHC
Ultrafiltration	19.4	12.2
Dialysis	6.4	5.0

Rat serum samples (n = 4 rats) were pooled and split into technical replicates (n = 5–6/method) for total steroid quantification, ultrafiltration, and equilibrium dialysis. DOC and progesterone were below the LLOQ in ultrafiltrates. Percent coefficient of variation (%CV) is shown. Abbreviations: DHC, 11-dehydrocorticosterone

**Fig 4:**
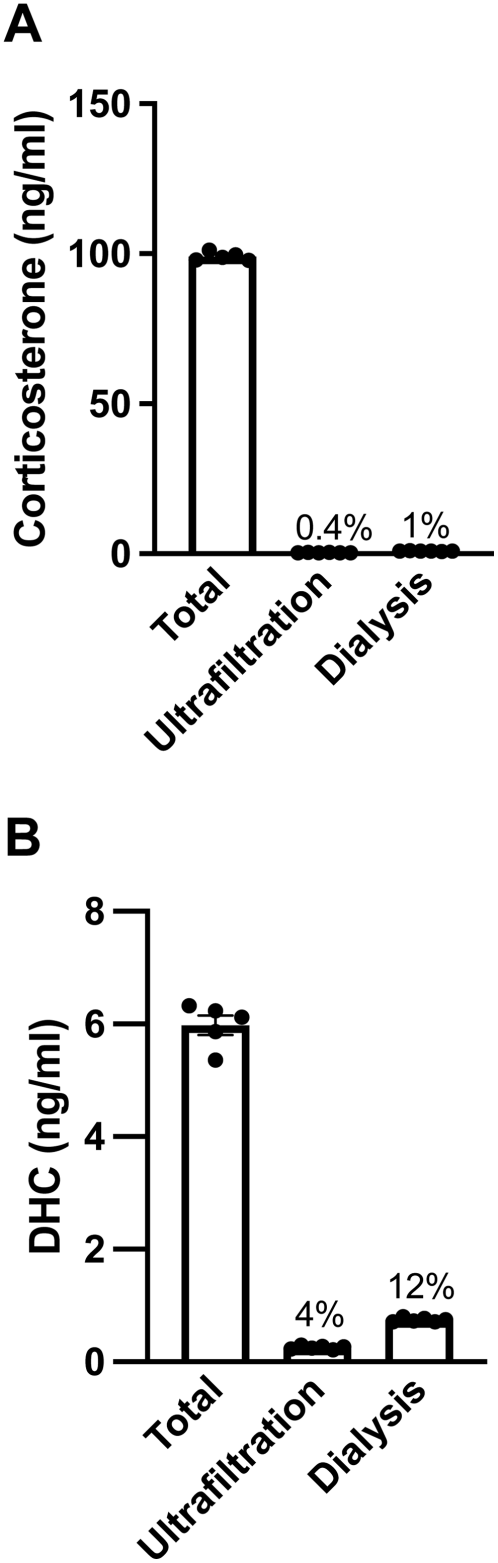
Free steroids measured via ultrafiltration and equilibrium dialysis in rat serum. (A) Corticosterone and (B) 11-dehydrocorticosterone (DHC) total and free levels (ng/ml). Rat serum samples (n = 4 rats) were pooled and then split into technical replicates (n = 5–6/method) for total steroid quantification, ultrafiltration, and equilibrium dialysis. Data are shown as mean ± SEM. The average percent free steroid is denoted above bars.

Using rat serum, free steroid levels (and the percent free steroid) were again similar with both methods but slightly lower with ultrafiltration than equilibrium dialysis (Fig 4). For example, the percent free corticosterone was 0.4% using ultrafiltration and 1% using equilibrium dialysis (Fig 4A). Again, the percent free DHC was greater than the percent free corticosterone with both methods. DOC and progesterone were below the LLOQ in ultrafiltrates but not dialysates [58].

### Study 4 – application of ultrafiltration to measure the effects of LPS in individual mice

**Fig 5:**
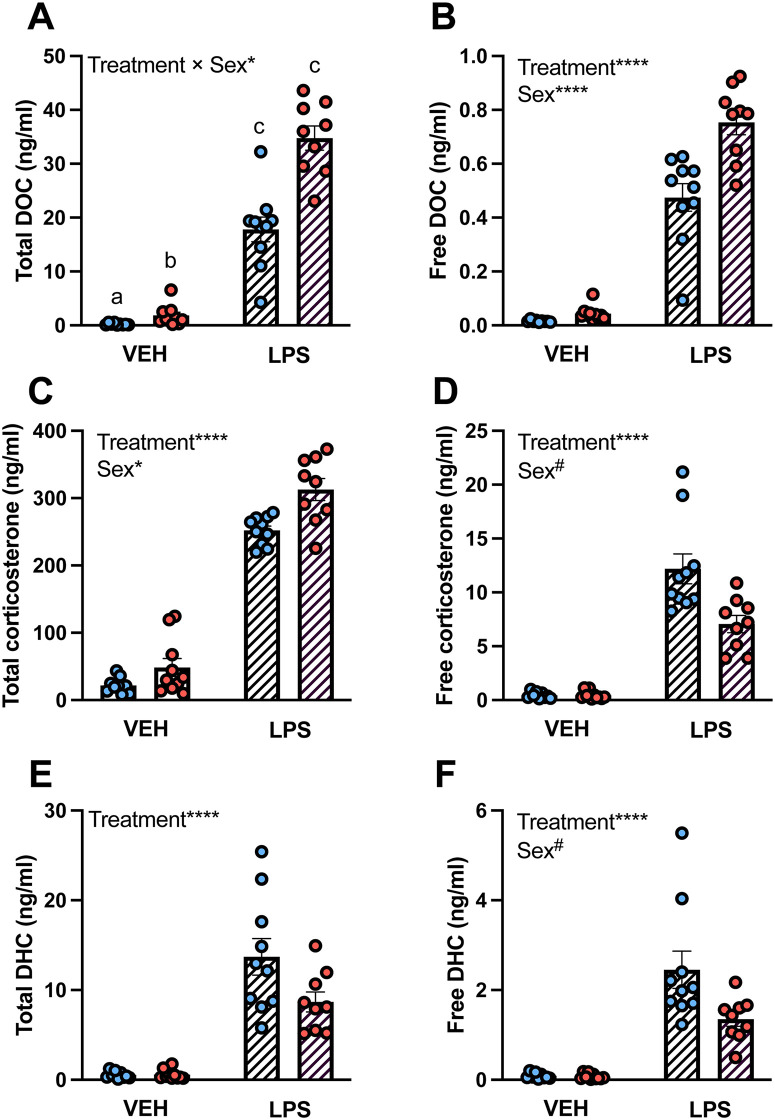
Total and free steroid levels measured via ultrafiltration in mice treated with vehicle or lipopolysaccharide. (A, B) 11-deoxycorticosterone (DOC), (C, D) corticosterone, and (E, F) 11-dehydrocorticosterone (DHC) total and free levels (ng/ml). Male (blue circles) and female (red circles) mouse serum samples were collected 4 hr after an i.p. injection of saline vehicle (VEH, open bars) or 50 μg/kg bw lipopolysaccharide (LPS, hatched bars). Serum samples from individual mice (n = 9–10/treatment/sex) were used for total steroid quantification and ultrafiltration. Data are shown as mean ± SEM. Data were analyzed by two-way ANOVAs. Significant main effects of Treatment and Sex and significant Treatment × Sex interactions are denoted. In cases of significant interactions, post hoc analyses were conducted using Tukey’s multiple comparisons tests and denoted by letters. #p ≤ 0.1, *p ≤ 0.05, ****p ≤ 0.0001.

Using serum samples from *individual* mice, we quantified total progesterone, DOC, corticosterone, and DHC levels in VEH- and LPS-treated mice. For total progesterone, there was a significant main effect of Treatment (F(1,34)=89.12, p < 0.0001), a significant main effect of Sex (F(1,34)=20.93, p < 0.0001), and a significant Treatment × Sex interaction (F(1,34)=50.01, p < 0.0001). Total progesterone levels were lower in VEH males than VEH females (p < 0.0001), lower in VEH males than LPS females (p < 0.0001), higher in LPS males than VEH females (p = 0.01), and were increased by LPS in males (p < 0.0001) [58]. For total DOC, there was a significant main effect of Treatment (F(1,34)=291.60, p < 0.0001), a significant main effect of Sex (F(1,34)=33.54, p < 0.0001), and a significant Treatment × Sex interaction (F(1,34)=6.25, p = 0.02). Total DOC levels were lower in VEH males than VEH females (p < 0.0001), lower in VEH males than LPS females (p < 0.0001), higher in LPS males than VEH females (p < 0.0001), and were increased by LPS in both sexes (p < 0.0001 in both cases) (Fig 5A). For total corticosterone, there was a significant main effect of Treatment (F(1,34)=191.0, p < 0.0001, VEH < LPS) and a significant main effect of Sex (F(1,34)=5.46, p = 0.03, M < F) (Fig 5C). For total DHC, there was a significant main effect of Treatment (F(1,34)=228.60, p < 0.0001, VEH < LPS) (Fig 5E).

Using 30 µl serum samples from individual mice and ultrafiltration, we quantified free progesterone, DOC, corticosterone, and DHC levels in the same VEH- and LPS-treated mice. Free progesterone values were below the LLOQ. For free DOC, there was a significant main effect of Treatment (F(1,34)=566.30, p < 0.0001, VEH < LPS) and a significant main effect of Sex (F(1,34)=31.68, p < 0.0001, M < F) (Fig 5B). For free corticosterone, there was a significant main effect of Treatment (F(1,34)=312.40, p < 0.0001, VEH < LPS) and a trend for a main effect of Sex (F(1,34)=3.19, p = 0.08, M > F) (Fig 5D). For free DHC, there was a significant main effect of Treatment (F(1,34)=247.10, p < 0.0001, VEH < LPS) and a trend for a main effect of Sex (F(1,34)=3.84, p = 0.06, M > F) (Fig 5F).

We quantified percent free DOC, corticosterone, and DHC in the same VEH- and LPS-treated mice (Table 4). Percent free DOC was significantly higher in VEH-treated males than LPS-treated males (t(8.21)=3.99, p = 0.004) but did not differ between VEH-treated females and LPS-treated females (t(9.25)=1.63, p = 0.14). As expected, percent free corticosterone was significantly lower in VEH-treated males than LPS-treated males (t(10.83)=4.76, p = 0.0006) and was also significantly lower in VEH-treated females than LPS-treated females (t(17)=4.34, p = 0.0004). Percent free DHC did not significantly differ between VEH-treated males and LPS-treated males (t(17)=1.15, p = 0.27), although there was a trend for a difference between VEH-treated females and LPS-treated females (t(17)=2.0, p = 0.06).

**Table 4:** Percent (%) free steroid levels in serum of individual mice treated with vehicle (VEH) or lipopolysaccharide (LPS).

A) Males			
**Treatment**	**DOC**	**Corticosterone**	**DHC**
VEH	10.2 ± 1.8	2.0 ± 0.2	16.3 ± 1.1
LPS	2.7 ± 0.2**	4.9 ± 0.6***	18.2 ± 1.2
**B) Females**			
**Treatment**	**DOC**	**Corticosterone**	**DHC**
VEH	4.2 ± 1.2	1.1 ± 0.2	12.5 ± 1.2
LPS	2.2 ± 0.1	2.2 ± 0.2***	15.9 ± 1.2

(A) Male and (B) female mouse serum samples were collected 4 hr after an i.p. injection of saline vehicle (VEH) or 50 μg/kg bw lipopolysaccharide (LPS). Serum samples from individual mice (n = 9–10/treatment/sex) were used for total steroid quantification and ultrafiltration. Data are shown as mean ± SEM. Data were analyzed using unpaired two-tailed t-tests. Significant differences between VEH- and LPS-treated mice are denoted. **p ≤ 0.01, ***p ≤ 0.001. Abbreviations: DOC, 11-deoxycorticosterone; DHC, 11-dehydrocorticosterone.

Correlations between total levels and free levels of DOC, corticosterone, and DHC levels in serum are presented in Table 5. Total levels and free levels of DOC were significantly positively correlated only in male LPS and female VEH groups. Total levels and free levels of corticosterone were significantly positively correlated in male and female VEH groups and the female LPS group – but not in the male LPS group. Total levels and free levels of DHC were significantly positively correlated in all groups.

**Table 5:** Correlations between total and free steroid levels in serum of individual mice treated with vehicle (VEH) or lipopolysaccharide (LPS).

Group		DOC	Corticosterone	DHC
Male VEH	*ρ*	.317	**.850**	**.933**
	p-value	.410	**.006**	**.001**
Male LPS	*ρ*	**.818**	−.309	**.879**
	p-value	**.006**	.387	**.002**
Female VEH	*ρ*	**.867**	**.891**	**.867**
	p-value	**.002**	**.001**	**.002**
Female LPS	*ρ*	.217	**.683**	**.800**
	p-value	.581	**.050**	**.014**

Male and female mouse serum samples were collected 4 hr after an i.p. injection of saline vehicle (VEH) or 50 μg/kg bw lipopolysaccharide (LPS). Serum samples from individual mice (n = 9–10/treatment/sex) were used. Spearman’ rho (ρ) and p-values are denoted. Abbreviations: DOC, 11-deoxycorticosterone; DHC, 11-dehydrocorticosterone.

## Discussion

We developed a protocol using Centrifree Ultrafiltration Devices and LC-MS/MS to measure free DOC, corticosterone, and DHC in 30 μl of mouse and rat serum. Then we applied this method to determine free glucocorticoid levels in VEH- and LPS-treated mice. Few studies have directly measured free steroids in mouse serum. This simple, robust, and rapid method should therefore be useful for measuring free levels of corticosterone and other steroids in small animals.

### Measuring multiple free glucocorticoids in a small volume of serum

In Study 1, we validated the use of ultrafiltration with as little as 30 μl of mouse serum. Overall, free DOC, corticosterone, and DHC can be measured in 30–150 μl of mouse serum, with an underestimation of free DHC when using 30 μl. We did not detect free progesterone (even with 150 µl), likely because 1) progesterone levels are far lower than corticosterone levels and 2) progesterone binds CBG with relatively high affinity [59].

This method can measure multiple free steroids in a small volume of serum. Free DOC and DHC in the circulation can enter tissues and be locally converted to corticosterone [60,61], and thus, free DOC and DHC should be measured in addition to free corticosterone. To our knowledge, no studies have directly measured free DOC or DHC levels in mice. LC-MS/MS allows simultaneous measurement of multiple steroids with high specificity and sensitivity [62]. Immunoassays for DOC and DHC have very limited availability and often lack sufficient specificity and sensitivity to measure the small amounts of free DOC and DHC [63,64]. Furthermore, when working with small animal models, such as mice and developing rats, the volume of blood that can be collected is limited [65], often providing researchers with less than 150 μl of serum. To our knowledge, no other method can measure free glucocorticoids in 30 μL of serum. Thus, our method is useful for measuring free corticosterone and other steroids in as little as 30 μl of serum.

### Ultrafiltration versus equilibrium dialysis for free glucocorticoid measurement

In Studies 2 and 3, we compared ultrafiltration and equilibrium dialysis and measured free DOC, corticosterone, and DHC in serum pools from VEH- and LPS-treated mice (Study 2) and from untreated rats (Study 3). Equilibrium dialysis is a well-established method to measure free steroid levels, but it requires ~24 hr and a larger volume of serum (200 + µl). First, in both studies, as expected, free GC levels are far lower than total GC levels with both methods. Second, in both studies, free GC levels are generally similar with both methods, although slightly lower with ultrafiltration than equilibrium dialysis (see below). Third, in Study 2, the effects of LPS are similar with both methods. Fourth, in both studies, relative differences across steroids (e.g., percent free corticosterone versus percent free DHC) are similar with both methods.

It is unclear why free steroid levels are slightly lower with ultrafiltration than equilibrium dialysis. It is possible that some free glucocorticoids were trapped in the dead space or adhered to the walls or filter of the ultrafiltration device [66]. Additionally, temperature fluctuations during ultrafiltration may affect CBG binding affinity, and thus, free glucocorticoid levels [42,43]. Furthermore, incomplete equilibration (i.e., serum not reaching 37°C) before centrifugation could alter CBG binding affinity. However, the magnitude of the difference in free steroid levels is small and consistent across groups, allowing ultrafiltration to be used for relative comparisons across groups within an experiment.

Both methods showed acceptable precision, although precision was lower with ultrafiltration. This is likely because equilibrium dialysis uses 6–7 × more serum than ultrafiltration (200 μl and 30 μl serum, respectively), thus increasing the amount of steroid in the sample for quantification by LC-MS/MS. In a similar vein, precision is lower for free DOC than free corticosterone, and this is likely because of the lower concentration of DOC.

Equilibrium dialysis is a well-known method to directly measure free corticosterone [67]. However, equilibrium dialysis requires a higher serum volume (200 + μl) than our ultrafiltration method, is more time-consuming, includes more steps, and is more prone to human error (e.g., contamination of dialysate with serum). Compared to equilibrium dialysis, ultrafiltration is faster, simpler, and requires less serum. However, a limitation of ultrafiltration is the higher cost of ultrafiltration devices compared to in-house equilibrium dialysis devices. Moreover, it remains difficult to detect free steroids with very low concentrations (e.g., free progesterone).

Ultrafiltration has advantages over estimation of free steroid levels using models. Estimates of free corticosterone from models might be inaccurate because CBG affinity for corticosterone is temperature-sensitive [42,43], other proteins (e.g., albumin) bind corticosterone, and other steroids (e.g., progesterone) compete with corticosterone for CBG binding. Overall, ultrafiltration combined with LC-MS/MS provides a robust, replicable, rapid, and straightforward method to directly quantify free glucocorticoid levels, addressing key limitations of existing methods, such as the difficulty of obtaining radiolabelled steroids, upon which many assays rely [44].

### LPS treatment increases free corticosterone levels in serum

In Study 4, we applied our novel method to measure free DOC, corticosterone, and DHC in serum from *individual* VEH- and LPS-treated adult mice, as a proof of principle. During an innate immune response, such as that elicited by LPS, CBG is cleaved, CBG-bound corticosterone is released, and the percent free corticosterone increases in circulation [18,30–34,36,37,68,69]. Here, LPS leads to varied increases in total and free DOC, corticosterone, and DHC levels. For instance, the percent of DOC that is free decreases, but the percent of corticosterone that is free increases, after LPS treatment. Furthermore, total corticosterone levels do not always correlate with free corticosterone levels, such as in male mice treated with LPS. Thus, it is not sufficient to measure total serum corticosterone levels and then infer free serum corticosterone levels for individual animals. The free steroid levels in Study 4 are consistent with those in Studies 1 and 2.

Several sex differences are apparent. First, as expected [22], total corticosterone levels are higher in females than males. In contrast, for free corticosterone levels, there is a non-significant trend for higher levels in males than females. Similarly, in rats, total corticosterone levels are higher in females than males, but free corticosterone levels at baseline are not different between the sexes [70], which might be explained by higher plasma CBG levels in females [70,71]. Second, total DOC levels were higher in females than males in the VEH-treated animals, and, similarly, free DOC levels were also higher in females than males. CBG binds DOC with lower affinity than corticosterone [22–27], and this might explain why the patterns for total and free DOC are more similar than those for corticosterone.

## Conclusions

We validated the use of a simple and rapid ultrafiltration procedure followed by a specific and sensitive LC-MS/MS assay to measure free DOC, corticosterone, and DHC in 30 μL of serum from mice and rats. We then applied this method to serum from VEH- and LPS-treated adult mice and observed the expected increases in total and free glucocorticoids and percent free corticosterone in LPS-treated mice. Future studies should investigate the effects of early-life stress on CBG and free glucocorticoid levels. This method also might be useful for measuring free glucocorticoids in other species, such as small songbirds [72,73], free levels of other steroids, such as testosterone [45,74–77], and correlations between free glucocorticoids and behaviour [78].

## Supporting information

S1 File Step-by-step protocol, also available on protocols.io. (PDF)
