# Supplementary material for: Direct measurement of free glucocorticoids in small volumes of mouse and rat serum using ultrafiltration and liquid chromatography-tandem mass spectrometry
Source: PLoS One. 2026 Jan 22;21(1):e0341089. doi: 10.1371/journal.pone.0341089 (PMC12826515; doi:10.1371/journal.pone.0341089)
Supplement: S1 File — (PDF) [file pone.0341089.s001.pdf]

Protocol to filter free steroids from serum using ultrafiltration and extract steroids via liquid-liquid extraction

RESERVED DOI:
10.17504/protocols.io.5qpvodkddg4o/v1

Anna Mazurenko[1,2], Melody Salehzadeh[2,3], Kiran K. Soma[1,2,3,4]

[1]Department of Psychology, The University of British Columbia, Vancouver, BC, Canada;
[2]Djavad Mowafaghian Centre for Brain Health, The University of British Columbia, Vancouver, BC, Canada;
[3]Department of Zoology, The University of British Columbia, Vancouver, BC, Canada;
[4]Graduate Program in Neuroscience, The University of British Columbia, Vancouver, BC, Canada

melody.salehzadeh

Protocol Info: Anna Mazurenko, Melody Salehzadeh, Kiran K. Soma . Protocol to filter free steroids from serum using ultrafiltration and extract steroids via liquid-liquid extraction. **protocols.io** **https://protocols.io/view/protocol-to-filter-free-steroids-from-serum-using-g8jqbzumx**

Created: August 26, 2025

Last Modified: August 27, 2025

Protocol Integer ID: 225616

Keywords: stress, cortisol, sepsis, protein binding, steroid profiling

Abstract

Direct measurement of free glucocorticoids in small volumes of mouse and rat serum using ultrafiltration and liquid chromatography-tandem mass spectrometry

Guidelines

Recommended schedule:
- Day 1: Prep and conduct ultrafiltration on serum samples
- Day 2: Prep and extract steroids from ultrafiltered serum samples using liquid-liquid extraction

Materials

Materials and equipment explicitly listed on these pages:
- Ultrafiltration device (#4104, Sigma-Aldrich, Oakville, Ontario, Canada)
- Bead ruptor (BR) tubes (Fisher Scientific, Cat # NC0934160) containing five 1.4 mm ceramic oxide beads per tube (2 BR tubes per serum sample: 1 for free glucocorticoids, 1 for total glucocorticoids)
- Avanti J-15R centrifuge (Beckman Coulter) with rotor adaptors that fit the ultrafiltration devices
- Water bath set to 37°C
- Bucket with wet ice
- Pipettes and appropriate tips (able to pipette 5 µl, ≥30 µl, 10 µl, 50 µl, and 1 mL)
- Red reservoir caps for ultrafiltration devices
- Collection tubes for ultrafiltrate
- Biohazard waste container for discarded ultrafiltration devices
- Standards and quality control samples as in Table 2 (steroid/glucocorticoid standards at pg/10 µl): Standard 1 = 0.4; 2 = 0.8; 3 = 2; 4 = 5; 5 = 12.5; 6 = 50; 7 = 100; 8 = 200; 9 = 500; 10 = 1000; 11 = 2000 pg/10 µl
- Quality control — Low concentration x3 = 2 pg/10 µl; High concentration x3 = 200 pg/10 µl
- Blanks: Blank x2; Double blank x2
- BR tubes for filtered and non-filtered serum aliquots (stored at -70°C)
- Oven (preheat to 60°C)
- 0.6 mL polypropylene microcentrifuge tubes (labelled to correspond with BR tubes)
- 2 mL glass LC-MS/MS vials (with glass inserts) corresponding to BR tubes
- Caps for LC-MS/MS vials
- 12×75 mm glass culture tubes (MeOH-rinsed and oven-dried) — prepare two distinct glass culture tubes per sample (labelled e.g., 1a, 2a,... and 1b, 2b,...). Cover with aluminum foil until use
- 20 mL scintillation vials and a Duran bottle for MilliQ water
- HPLC-grade Ethyl Acetate, Methanol
- Fresh Milli-Q water
- Ability to prepare 50% MeOH and 25% MeOH solutions in rinsed 20 mL scintillation glass vials
- Small water bath (preheat to 65°C in fume hood)
- Vortex mixer
- Homogenizer/bead ruptor capable of 4 m/s
- Centrifuge capable of 16,100 g
- Glass culture tubes labelled to collect supernatant (e.g., 1a, 2a, 3a, ...)
- Aluminum foil for covering glass tubes
- Manifold with N2 gas supply (nitrogen gas manifold for drying Ethyl Acetate under N2)
- Tools for transferring ~150 µl to LC-MS/MS vials (pipettes/tips)
- Storage freezer set to -20°C (for extracted samples prior to injection)

Safety warnings

Safety and caution notes present on these pages:
- Keep serum samples at physiological body temperature (i.e., 37°C for mice) during processing to preserve free glucocorticoid distribution.
- It will take at least 30 min for the centrifuge to stabilize at 37°C — ensure devices are prewarmed before use.
- Discard used ultrafiltration devices in biohazard waste.
- Handle samples and disposables using appropriate biosafety precautions.
- Note to leave sufficient serum for non-filtered aliquots (5 µl) before filtering.
- Only work with ethyl acetate (highly volatile) in the fume hood using appropriate personal protective equipment (PPE)
- Work quickly to minimize ethyl acetate evaporation when added to BR tubes.
- Follow institutional safety procedures when working with organic solvents (e.g., Ethyl Acetate, Methanol)
- Use caution when operating the N2 gas manifold and ensure proper ventilation and secure connections to prevent leaks.
- Cap glass LC-MS/MS vials carefully and ensure there are no air bubbles in the samples prior to storage/injection.

Before start

Only use HPLC- or LCMS- grade reagents.
Only use fresh MilliQ water (i.e., do not used water stored for a long time, or in container that has been opened/closed multiple times).
Only use polypropylene-grade plastics.
Rinse all glassware (except for LC-MS/MS vials/inserts) with HPLC-grade methanol two times.

Preparation for ultrafiltration

1 Label 1 ultrafiltration device (#4104, Sigma-Aldrich, Oakville, Ontario, Canada) per serum sample

2 Label 1 set of bead ruptor (BR) tube (Fisher Scientific, Cat # NC0934160) containing 5 1.4mm ceramic oxide beads for all serum samples for **free** steroid measurement

3 Label second set of bead ruptor (BR) tube (Fisher Scientific, Cat # NC0934160) containing 5 1.4mm ceramic oxide beads for all serum samples for **total** steroid measurement

4 Obtain ice bucket with wet ice

5 Thaw serum samples gently on wet ice

6 Prewarm centrifuge (Avanti J-15R, Beckman Coulter) **and** rotor adaptors (to fit the ultrafiltration devices) to 37°C (physiological body temperature of mice). You may need to spin centrifuge (i.e., 2000 g) for device to warm, follow manufacturer's instructions.

7 Warm water bath to 37°C (physiological body temperature of mice)

Ultrafiltration protocol

1h 10m

8 Once serum samples are thawed, place serum samples in water bath at 37°C (physiological body temperature of mice) for 10 min

10m

9 Remove red reservoir caps off all labelled ultrafiltration devices

10 Pipette at least 30 µl serum of each sample into its respective ultrafiltration device, ensuring that you keep >5 µl of serum for total steroid measurement

11 Cap ultrafiltration devices

12 Spin ultrafiltration devices at 2000 g at 37°C (physiological body temperature for mice) for 1 hr

1h

13 While serum samples are being centrifuged, add 5 µl of remainder of each serum sample to respective labelled BR tubes labelled for total steroid measurement (i.e., non-filtered serum samples)

14 Store non-filtered serum samples for total steroid measurement (5 µl) at -70°C until extraction

15 Store any remaining serum that was not used for free and total serum measurement in -70°C

16 After spinning ultrafiltration devices for 1hr, transfer the entire ultrafiltrate from filter collection tubes to corresponding new BR tubes (i.e., filtered serum samples)

17 Store filtered serum samples for free steroid measurement (entire volume) at -70°C until extraction

18 Discard used ultrafiltration devices in appropriate waste according to institutional policies (e.g., biohazard waste)

Preparation for liquid-liquid extraction

10m 2s

19 Preheat oven to 60°C

20 Obtain and label 1 set of bead ruptor (BR) tube containing 5 1.4mm ceramic oxide beads per standard curve/quality control/blank/double blank samples (filtered/non-filtered serum samples should already be in BR tubes)

21 Obtain 2 sets of 12×75 mm glass culture tubes

22 Add 1mL of HPLC-grade methanol to all 12×75 mm glass culture tubes

23 Vortex 12×75 mm glass culture tubes containing 1mL HPLC-grade methanol to rinse glassware for 2s

2s

24 Discard HPLC-grade methanol from all 12×75 mm glass culture tubes

25 Repeat steps 22-24 to rinse 12×75 mm glass culture tubes a second time

26 Place rinsed 12×75 mm glass culture tubes (rinsed twice) upside down in metal tube rack

27 Dry rinsed 12×75 mm glass culture tubes (rinsed twice) upside down in oven at 60°C for 10min

10m

28 Invert rinsed and dried 12×75 mm glass culture tubes to be right-side up in tube rack

29 Label first set of rinsed and dried 12×75 mm glass culture tubes 1a, 2a, 3a, ..., *n*a

30 Label second set of rinsed and dried 12×75 mm glass culture tubes 1b, 2b, 3b, ..., *n*b

31 Cover rinsed and dried 12×75 mm glass culture tubes with aluminum foil under ready to use

32 Label sealed 0.6 mL polypropylene microcentrifuge tubes to correspond with all BR tubes

33 Label 2 mL glass LC-MS/MS vials to correspond with all BR tubes

34 Place glass inserts into glass LC-MS/MS vials

35 Cover glass LC-MS/MS vials with aluminum foil to store until ready to use

36 Rinse 2 20 mL scintillation vials, a 500mL Duran bottle for MilliQ water, and 3 500mL beakers, with HPLC-grade methanol

37 Discard of methanol glassware rinse waste

38 Repeat steps 36-37 to rinse glassware twice

39 Dry rinsed 20mL scintillation vials, 500mL Duran bottle, 3 500mL beakers (e.g., in fume hood)

40 Retrieve 500mL of fresh MilliQ water in rinsed 500mL Duran bottle

41 Preheat water bath to 65°C in the fume hood

42 Prepare 10 mL of 50% HPLC-grade methanol (diluted with MilliQ water) in rinsed 20 mL scintillation glass vial

43 Prepare 20 mL of 25% HPLC-grade methanol (diluted with MilliQ water) in rinsed 20 mL scintillation glass vial

44 Thaw standard curve standards (0.02 pg/µL - 400 pg/µL, depending on study) and internal standard mix (10 pg/µL) (stored at -20°C) on bench top and allow to come to room temperature

45 Thaw filtered, and non-filtered serum samples (in BR tubes) for free and total steroid measurement, respectively (stored at -70°C) gently on wet ice

Liquid-liquid extraction protocol

18m 37s

46 Add 10 µL of standard curve standards (0.02 pg/µL - 400 pg/µL, depending on study) to respective BR tubes

47 Add 10 µL of low concentration (0.2 pg/µL) and high concentration (20 pg/µL) quality controls to respective BR tubes

48 Add 10 µL of HPLC-grade 50% methanol into blank BR tubes to make volumes equal

49 Add 50 µL internal standard mix (10 pg/µL) to all BR tubes except double blank BR tubes

50 Add 60 µL of HPLC-grade 50% methanol into double blank BR tubes to make volumes equal

51 Add 1 mL HPLC-grade Ethyl Acetate into all BR tubes (standard curve, blanks, double blanks, quality controls, samples) in fume hood

52 Vortex all BR tubes for 2 sec

2s

53 Homogenize samples at 4 m/s for 30 sec using a bead mill homogenizer (i.e., Fisherbrand™ Bead Mill 24 Homogenizer, Cat # 15-340-163) 30s

54 Centrifuge samples at 16,100 g for 5 min at room temperature 5m

55 Remove 1000 µl of supernatant and place in respective 12×75 mm glass culture tubes (i.e., glass culture tubes labelled 1a, 2a, 3a, ..., *n*a)

56 Add 500 µl MilliQ water to all samples

57 Vortex all samples for 5 sec

58 Centrifuge samples at 3200 g for 2 min at room temperature 2m

59 Carefully remove top liquid layer (ethyl acetate) from samples (without disturbing tissue pellets at the bottom) and transfer to respective new glass culture tubes (e.g., tubes labelled 1b, 2b, 3b, ..., *n*b), in a fume hood

60 Discard the glass culture tubes containing MilliQ water waste

61 Dry samples (in ethyl acetate in glass culture tubes) in water bath at 65°C for 8 min with N_2 gas, in a fume hood 8m

62 Resuspend dried samples in glass culture tubes with 200 µl 25% HPLC-grade methanol

63 Vortex samples in glass culture tubes for 5 sec to ensure resuspension 5s

64 Centrifuge samples in glass culture tubes at 3200 g for 1 min at room temperature 1m

65 Transfer sample (whole volume) to respective 0.6 mL polypropylene microcentrifuge tubes

66 Centrifuge samples in 0.6 mL polypropylene microcentrifuge tubes at 16,100 g for 2 min at room temperature 2m

67 Transfer 150 µl of supernatant to respective glass LC-MS/MS vial glass inserts

68 Cap glass LC-MS/MS vials and ensure no air bubbles in the samples

69 Store samples in glass LC-MS/MS vials in -20°C until injection into mass spectrometer (MS)

70 Inject sample into LC-MS/MS system according to manufacturer's guidelines (in our case, we injected 35 µl of each sample into LC-MS/MS)

Acknowledgements

Corresponding author/contact information:
Anna Mazurenko
annamaz@student.ubc.ca
https://orcid.org/0009-0009-8851-5335
